# ﻿First contribution to the genera *Branchiobaetis* and *Megabranchiella* (Ephemeroptera, Baetidae) in China, with descriptions of two new species

**DOI:** 10.3897/zookeys.1216.129803

**Published:** 2024-10-23

**Authors:** Xiaoli Tong, Zhiheng Zhou, Bangyi Wu

**Affiliations:** 1 Department of Entomology, College of Plant Protection, South China Agricultural University, Guangzhou 510642, Guangdong Province, China South China Agricultural University Guangzhou China

**Keywords:** Baetid, mayflies, new record, Southeast Asia, subtropical China

## Abstract

*Branchiobaetis* Kaltenbach, Kluge & Gattolliat, 2022 and *Megabranchiella* Phlai-ngam & Tungpairojwong, 2022 (Ephemeroptera: Baetidae) are newly recorded in China. Two new species, *Branchiobaetisborealis***sp. nov.** based on larval stage and *Branchiobaetismegasinus***sp. nov.** based on larval and imaginal stages associated by laboratory rearing, are described. *Megabranchiellalongusa* Phlai-ngam & Tungpairojwong, 2022, previously only distributed from Thailand, is recorded from China for the first time.

## ﻿Introduction

The family Baetidae is ubiquitous in freshwater habitats with the highest species diversity amongst mayflies, often being a major benthic component of freshwater ecosystems (Barber-James et al. 2008; Jacobus et al. 2019). It is almost worldwide distributed but is mostly diversified in the tropics (Gattolliat and Nieto 2009; Kaltenbach et al. 2022a, b). Southeast Asia, known for its expanses of tropical rainforests and river systems representing one of the most biologically diverse ecosystems on the Earth, harbours a unique and diverse mayfly fauna, which is one of the most species-rich hotspots of mayflies at the global scale (Gattolliat and Nieto 2009; Kaltenbach et al. 2023a). For a long time, however, systematics of mayflies (Baetidae in particular) has received little attention by taxonomists compared with those from Europe and North America. Although many Ephemeroptera taxonomists, such as Ulmer (1939), Kimmins (1947), Gillies (1949, 1951), and Müller-Liebenau (1980, 1981, 1984a, b) have made great contributions to the study of baetid fauna in the region, the knowledge of the baetid systematics is still sparse (Gattolliat and Nieto 2009), and baetid species diversity of Southeast Asia is under severe threat of extinction because of rapid economic development and urbanization. Encouragingly, significant progress has been made in baetid systematics in the region during the past decade or so. A large number of new genera and species of Baetidae have emerged like mushrooms after spring rain in Southeast Asia and its neighbouring areas. For example, eight new genera were established, *Asiobaetodes* Gattolliat (in Gattolliat 2012), *Acerobiella* Gattolliat (in Gattolliat 2012), *Procerobaetis* Kaltenbach & Gattolliat (in Kaltenbach et al. 2020b), *Cymbalcloeon* Suttinun, Gattolliat & Boonsong (in Suttinun et al. 2020), *Philibaetis* Kaltenbach & Gattolliat (in Kaltenbach et at. 2021), *Branchiobaetis* Kaltenbach, Kluge & Gattolliat (in Kaltenbach et al. 2022b), *Megabranchiella* Phlai-ngam & Tungpairojwong (in Phlai-ngam et al. 2022), *Arcobaetis* Kaltenbach, Kluge & Gattolliat (in Kaltenbach et al. 2023a), and dozens of new baetid species were described (e.g. Tong and Dudgeon 1999, 2021; Kluge and Novikova 2011, 2017; Gattolliat 2012; Shi and Tong 2014, 2015a, b, 2019; Tong et al. 2014; Kaltenbach and Gattolliat 2018, 2019, 2020, 2021, 2023; Kaltenbach et al. 2020a, 2023b; Kluge and Suttinun 2020; Suttinun et al. 2021, 2022; Li et al. 2023).

The baetid fauna of South China and Southwest China (e.g., Guangdong, Hong Kong, Hainan, Guangxi, Yunnan) shows important affinities with those of the mainland Southeast Asia and even share many baetid taxa with the insular Southeast Asia (Tong and Dudgeon 2002; Shi and Tong 2014, 2015a, b,^2019^). In our present surveys, two genera of Baetidae, formerly described only from Southeast Asia, are newly recorded from China, i.e., the genus *Branchiobaetis*, recently established by Kaltenbach et al. (2022b), is a small genus with seven species and these are previously known only from the archipelagic Southeast Asia (Indonesia, Malaysia, and Philippines); the genus *Megabranchiella* was erected by Phlai-ngam et al. (2022) from Thailand with two species, of which *Megabranchiellalongusa* Phlai-ngam & Tungpairojwong, 2022 is recorded from China for the first time. Here, we describe two new species of *Branchiobaetis* found from Guangdong, Hong Kong, Yunnan, China, and report the new record of *M.longusa* from Yunnan, China.

## ﻿Materials and methods

The larvae were collected with a D-frame net and then placed into vials containing 90% ethanol in the field, while some living mature larvae with black wing pads were transported to the laboratory in a plastic container containing stream water for rearing. Each reared subimago and imago together with its final instar exuviae and subimaginal skin were stored in a single vial containing 90% ethanol. The larvae were dissected in ethanol under a stereomicroscope, with subsequent mounting on slides by Hoyer’s solution. Photographs were taken using a Canon EOS 5D Mark IV camera with MP-E 65 mm macro lens and the microscope with a digital camera attached. Water physicochemical parameters were measured by portable multi-parameter meter (Hach sensION™156) in the field. The distribution map was downloaded from the National Platform for Common GeoSpatial Information Services (https://www.tianditu.gov.cn/). Type specimens have been deposited in the Insect Collection, South China Agricultural University (SCAU), Guangzhou, China.

## ﻿Results

### ﻿ Branchiobaetis borealis sp. nov.

Taxon classificationAnimaliaEphemeropteraBaetidae

B2506040-5F50-538B-811A-36BB58CAF7A4

https://zoobank.org/7E13C47C-A248-40C6-A77E-8F0BBBAEB188

**Figure 1.**
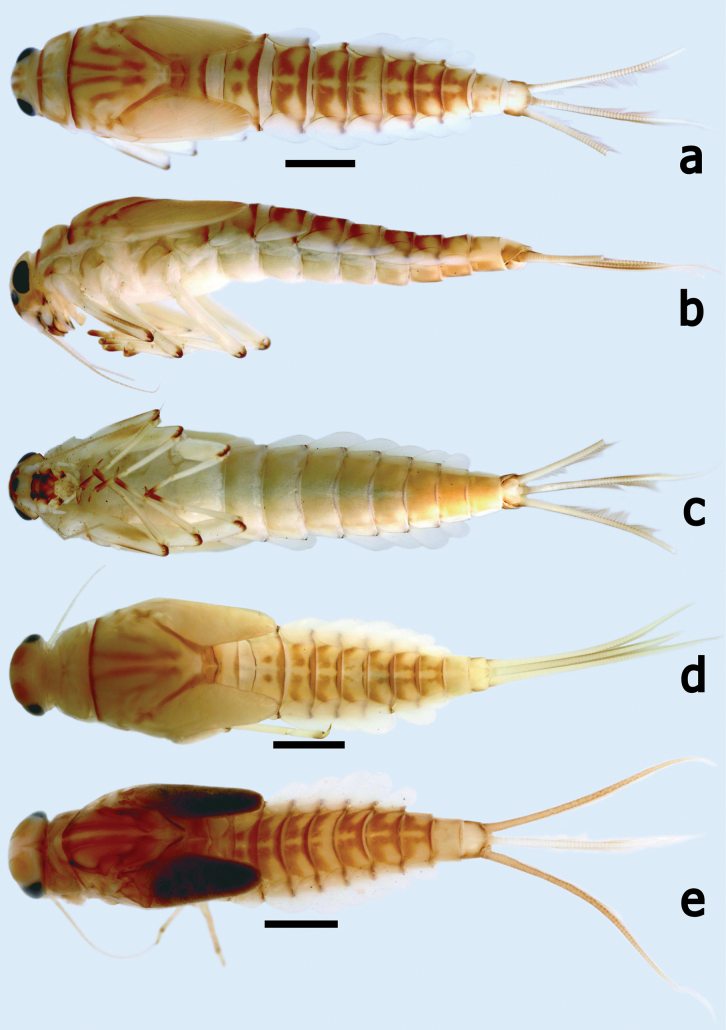
*Branchiobaetisborealis* sp. nov. Larval habitus **a** female larva (dorsal view) **b** female larva (lateral view) **c** female larva (ventral view) **d** male larva (dorsal view) **e** final instar male larva (dorsal view). Scale bars: 1.0 mm.

**Figure 2.**
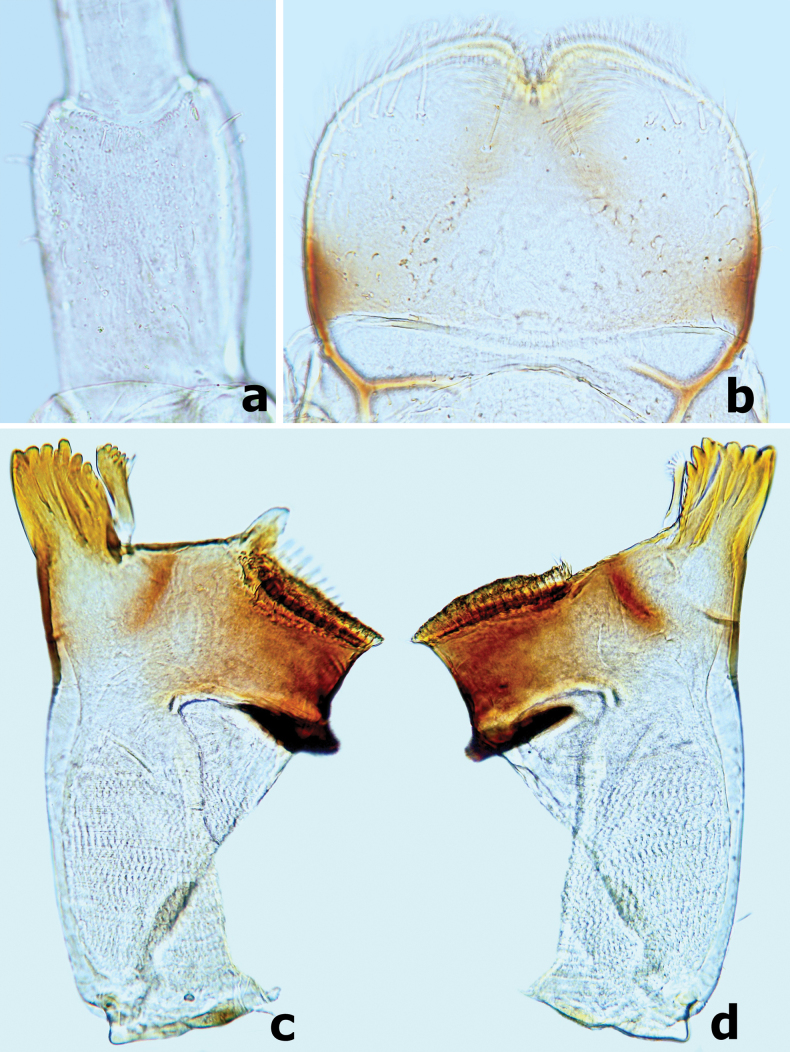
*Branchiobaetisborealis* sp. nov. **a** antennal pedicel **b** labrum **c** left mandible **d** right mandible.

**Figure 3.**
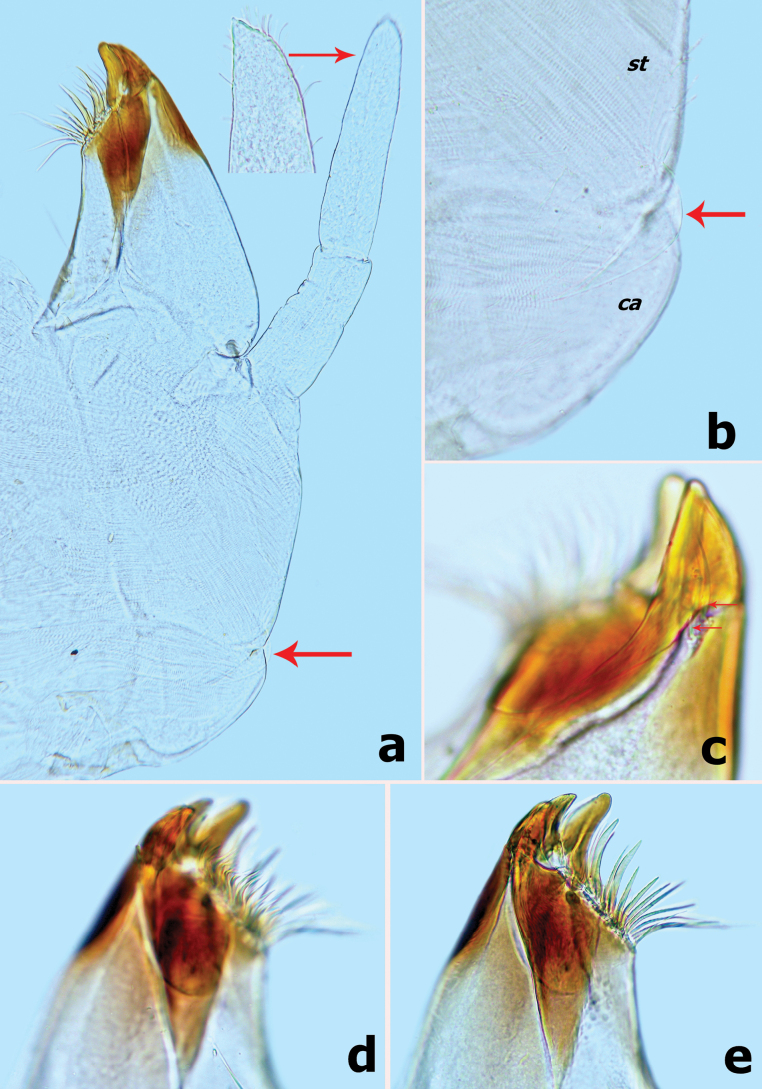
*Branchiobaetisborealis* sp. nov. **a** maxilla **b** accessory gill between stipes and cardo of maxilla **c–e** apex of maxilla. Abbreviations: st: stipes, ca: cardo.

**Figure 4.**
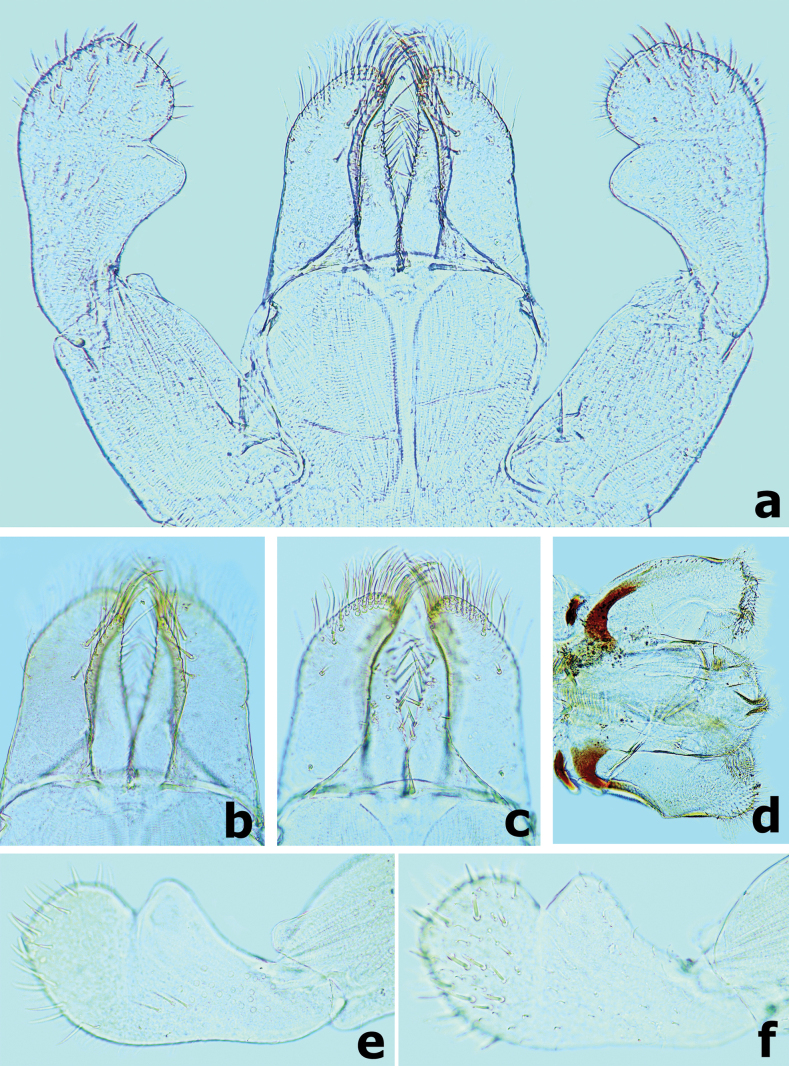
*Branchiobaetisborealis* sp. nov. **a** labium **b** glossae & paraglossae (dorsal view) **c** glossae and paraglossae (ventral view) **d** hypopharynx and superlinguae **e** segments II & III of labial palp (dorsal view) **f** segments II and III of labial palp (ventral view).

**Figure 5.**
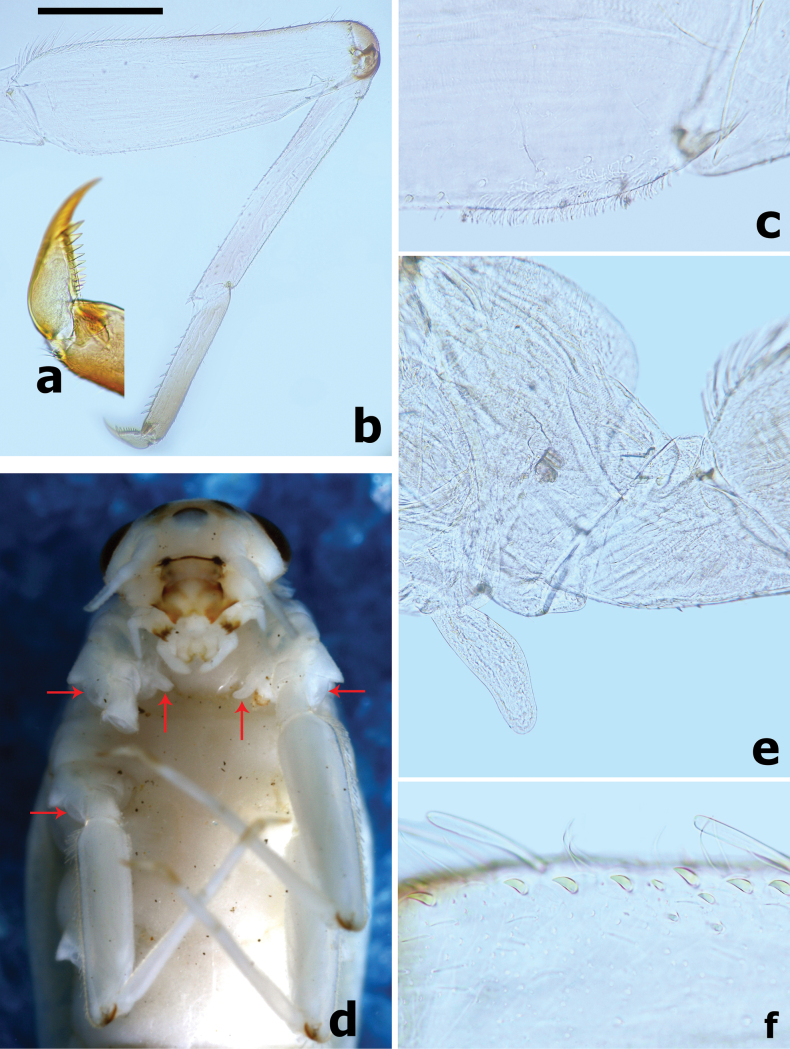
*Branchiobaetisborealis* sp. nov. **a** claw **b** foreleg **c** villopore of femur **d** accessory gills (vertical arrows) between coxa and prosternum on foreleg and bubble-like membranous swellings (horizontal arrows) **e** detail of accessory gill on foreleg **f** distally curved hook-like setae on dorsal margin of femur. Scale bar: 0.5 mm.

**Figure 6.**
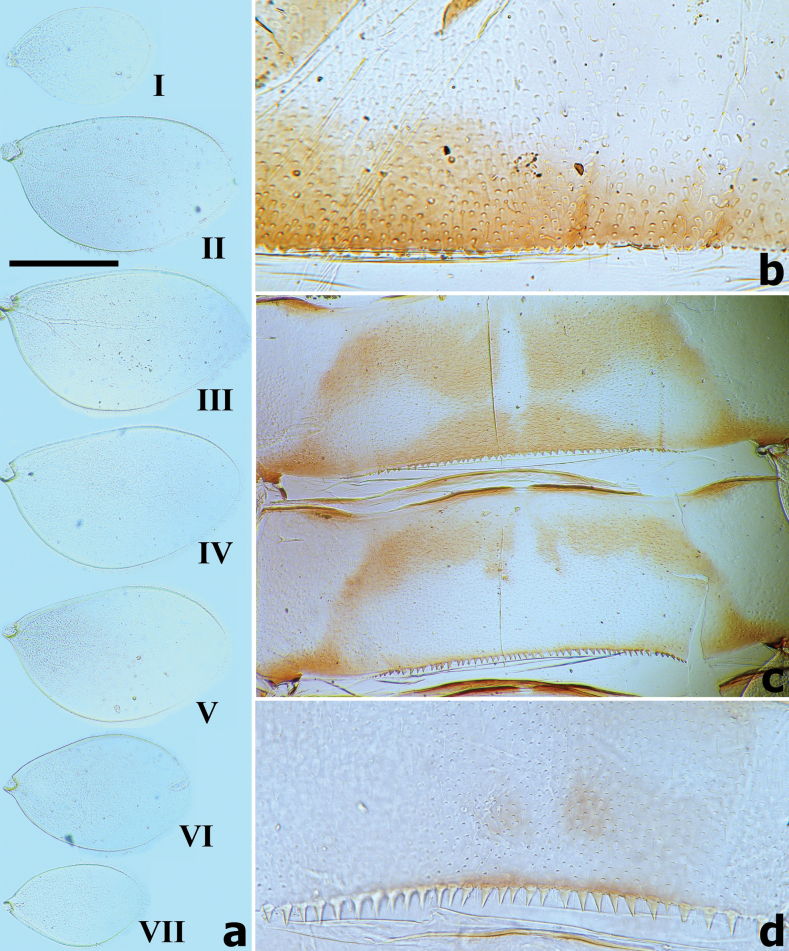
*Branchiobaetisborealis* sp. nov. **a** gills I–VII **b** posterior margin of abdominal tergite I **c** posterior margin of abdominal tergites IV–V **d** posterior margin of abdominal tergite IX. Scale bars: 0.5 mm.

**Figure 7.**
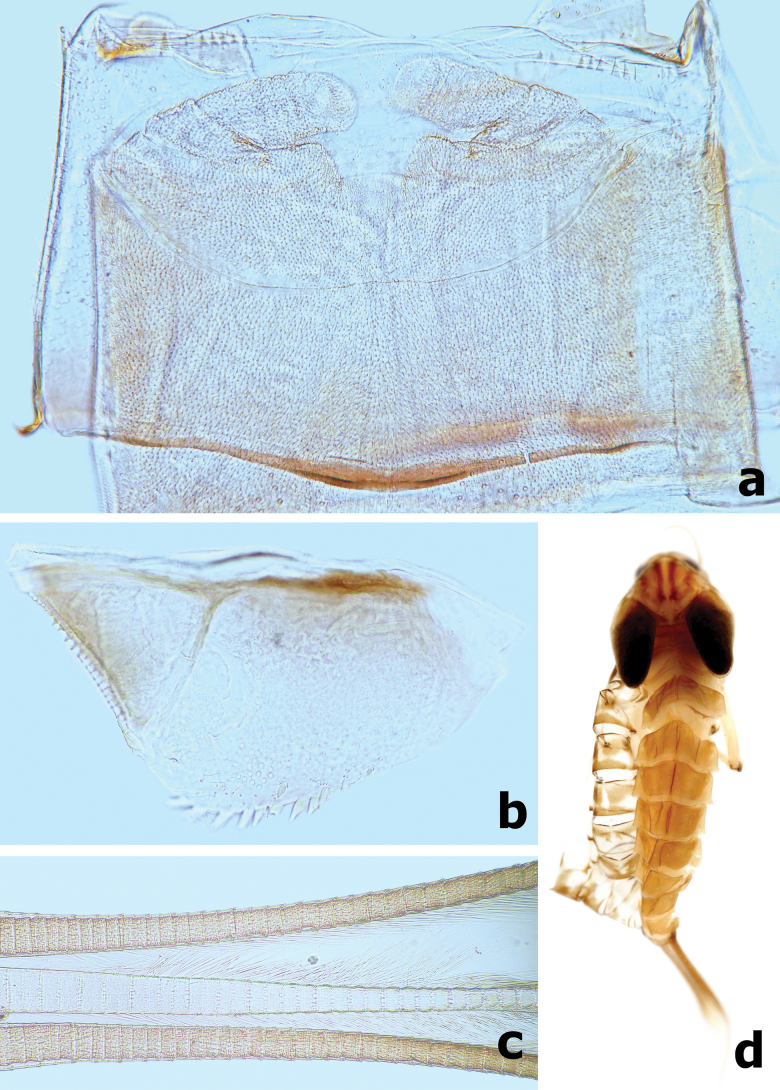
*Branchiobaetisborealis* sp. nov. **a** gonostyli bud under cuticle of last instar male larva **b** paraproct **c** part of caudalii **d** subimaginal female extracted from last instar larva before emergence.

Figs 1 , 2 , 3 , 4 , 5 , 6 , 7

#### Type material.

***Holotype*.** China • male larva in alcohol (mature); Yunnan, Lushui City, Chengan Town, a tributary of the Nujiang River (26.2605°N, 98.8792°E, altitude 1036 m); 21.iii.2019; leg. Xiaoli Tong. ***Paratypes*** (in alcohol): • 41 mature larvae (2 on slide), locality and date as holotype, leg. Xiaoli Tong, Lin Hong, Jian Jiang • 10 larvae (1 on slide); Yunnan, Weixi County, Tacheng Town, Lapu River (a tributary of the Jinsha River, 99.3507°E, 27.5728°N, altitude 2523 m); 8.xi.2018; leg. Xiaoli Tong, Lin Hong, Haoyang Chen • 13 larvae (1 on slide), Yunlong County, Caojian Town, a tributary of the Lancang River (25.6339°N, 99.1123°E, altitude 1824 m); 23.iii.2019; leg. Xiaoli Tong, Lin Hong, Jian Jiang.

#### Description.

Mature larva (Fig. 1a–e). Body length (mm): female 7.2–8.5, male larvae slightly shorter than female, 6.0–7.5; antenna 2.0–3.0; cerci 3.0–4.0, paracercus ~ 3/4 length of cerci.

***Cuticular colouration*.** Body mainly creamy yellow with brown maculae dorsally. Vertex and pronotum creamy yellow with irregular brown marks, meso- and metanotum creamy yellow with longitudinal brown streaks. Antennal scape and pedicel mainly off-white, flagellum pale brown. Femur of foreleg mainly off-white with dark brown apex and brown streaks along dorsal and ventral margins; tibia off-white; tarsus off-white with apical 1/2 dark brown; midlegs and hindlegs similar to forelegs in colour pattern. Abdominal tergites cream yellow with contrasting brown maculae as in Fig. 1a–e, tergite hypodermal colour uniformly without maculae or pigmentation (Fig. 7d); sternites with cream shading to pale brown backwardly. Gills white with transparent main trunk and branches of tracheae. Caudalii cream to yellow-brown with brown primary swimming bristles.

***Precursors of turbinate eyes*** in last instar male larvae normal, without elevated area with well-expressed facets.

***Antenna*** (Fig. 1b–e). Antenna ~ 3–4× head width; scape smooth with fine setae sparsely; pedicel surface with fine setae and one row of tiny, rounded setae along distal margin, inner margin with tiny, triangular denticles distolaterally (Fig. 2a).

***Labrum*** (Fig. 2b) nearly rectangular, width/length ratio ~ 1.6; anterior margin bordered with long and feathered setae and a deep notch; dorsally with submedial pair of long, robust bristles and submarginal arc of ~ 5 long, robust bristles on each side of midline, several fine setae scattered proximally; ventral surface with dense, fine setae medially and 6–8 short, pointed setae laterally and disto-laterally.

***Left mandible*** (Fig. 2c). Incisor and kinetodontium fused; incisor with three denticles, kinetodontium with three main denticles decreasing in length and one additional minute denticle between incisor and kinetodontium; prostheca robust, apex with five or six blunt denticles and two or three slender, pointed denticles; margin between prostheca and mola straight; apex of mola without tuft of setae.

***Right mandible*** (Fig. 2d). Incisor and kinetodontium fused; incisor with three denticles; kinetodontium with four denticles, inner margin of innermost denticle with three or four small denticles; prostheca slender with slightly wider base, apex toothbrush-like, with many sharp denticles on inner margin; margin between prostheca and mola slightly concave; apex of mola with a tuft of straight setae.

***Hypopharynx and superlinguae*** (Fig. 4d). Lingua subequal to superlinguae in length, with numerous fine setae apically. Superlinguae distally rounded with numerous fine setae along apical margin.

***Maxilla*** (Fig. 3a). Galea-lacinia with three robust canines; crown of galea-lacinia with one regular row of 10–13 medium-size arcuate, simple setae (Fig. 3d), second row composed of three dentisetae and a row of 7–9 elongated pectinate setae (Fig. 3e), 1^st^ dentiseta robust, canine-like with wide base, other dentisetae slender, bifid, and pectinate; ventrally with two simple, apical setae under canines (Fig. 3c). Medially with one row of five or six long, simple setae and one spine-like seta perpendicular to lacinia margin. Maxillary palp 2-segmented, longer than galea-lacinia, segment I shorter than segment II, apex of segment II with single tiny scale on small cone-shaped projection (Fig. 3a). Small tongue-like accessory gill located on outer side of the articulation between stipes and cardo (Fig. 3a, b).

***Labium*** (Fig. 4a). Glossae shorter and narrower than paraglossae, triangular with wide base, narrowing toward apex, inner margin of glossae with 10–12 spine-like, simple setae and outer margin with six or seven long, simple setae, apex with three robust setae; paraglossae with three rows of long, robust, distally pectinate and curved setae distoventrally and one short, simple seta in proximolateral area (Fig. 4c); dorsal surface on distal 1/2 with one longitudinal row of five or six long, robust, spine-like setae near inner margin (Fig. 4b); labial palp 3-segmented; segment I slightly shorter than segments II and III combined, with many micropores dorsally; segment II triangular with distinct protuberance apico-laterally, ~ 1.3× wider than base of segment III, dorsal surface with one longitudinal row of three or four medium spine-like simple setae and many micropores (Fig. 4e); segment III similar to asymmetrical onion-shaped dome, dorsally with pointed simple setae near apex, ventral surface covered with many blunt pointed spatulate setae accompanied by fine setae (Fig. 4f).

***Hind wing pads*** well developed.

***Forelegs*** (Fig. 5b). Ratio of foreleg segments (femur to claw) 6.8:5.4:3.0:1.0. *Femur*. Length ~ 3.4× maximum width. Dorsal surface covered with many small oval spatulate setae accompanied by fine setae; ventral face with sparse, small, spatulate setae and fine setae. Outer margin with two rows of different type setae: one row of 13–17 medium-sized, robust, clavate setae and proximal row of 17–22 long, slender setae; additional row of small, teardrop-shaped, distally curved, hook-like setae along dorsal margin (Fig. 5f); ventral margin with small, apically pointed or rounded, spatulate setae basally. Apex rounded with short, stout setae anteriorly, dorsoapical setal patch formed by two stout clavate setae. Villopore present and well developed (Fig. 5c). A transparent finger-like accessory gill on inner side of coxal articulation (between coxa and prosternum) (Fig. 5d, e); hyaline bubble-like membranous swelling between coxa and trochanter (Fig. 5d). *Tibia*. Outer margin with one row of fine setae and several small, curved hook-like setae; ventral margin with row of short, stout spine-like setae. Tibio-patellar suture present. Both surfaces covered with small, apically pointed or rounded, spatulate setae alternating with hair-like setae. *Tarsus*. Outer margin with fine setae and one row of small, distally curved hook-like setae; ventral margin with one row of ~ 10 stout spine-like pointed setae increasing in length towards apex; both surfaces covered with small, spatulate setae alternating with hair-like setae. *Claws* hooked (Fig. 5a), with one row of ~ 10 acute teeth, subapical setae absent.

***Middle and hind legs*** similar to foreleg in structure except for lacking the finger-like accessory gills on base of coxa and the villopore larger and more obvious than that of forelegs.

***Abdominal tergites and sternites*** both densely covered with crescent-shaped scale bases, several triangular spatulate setae, and hair-like setae. Posterior margins of tergites I–X with triangular spines increasing in length from I to X (Fig. 6b–d). Posterior margins of sternites I–IX smooth medially, but with row of dentate protuberances laterally (as Fig. 14b).

***Gills*** (Fig. 6a) present on segments I–VII and well tracheated with a transparent main trunk and branches of tracheae, ratio of gill length from I–VII = 1.1:1.7:1.9:1.8:1.7:1.3:1.0. External margins of all gills with small denticles intercalating fine hair-like setae, without any marginal spines or spatulate setae.

***Paraproct*** (Fig. 7b). Surface scattered with many micropores and hair-like setae, two or three small oval spatulate setae present near posterior margin; posterior margin with nine or ten triangular spines; surface of cercotractor smooth, with 19–22 spines marginally.

***Caudalii*** (Fig. 7c). Cerci with a row of swimming bristles on inner side of intersegment, each segment with row of pointed spines distally; paracercus with swimming bristles on both sides of intersegment.

***Gonostyli bud*.** Subimaginal gonostyli folded under cuticle of last instar larvae, segments II and III sharply bent towards middle (Fig. 7a).

#### Etymology.

The specific epithet *borealis* is the Latin masculine adjective, meaning “northern”, referring to the fact that this new species may represent the northernmost distribution of the genus in the Oriental Region.

#### Distribution

**(Fig. 23).** China: Yunnan (Lushi, Weixi, and Yunlong).

#### Larval habitat

**(Fig. 22b).***Branchiobaetisborealis* sp. nov. was found in moderately rapid to swift unshaded streams with gravel substrates at altitudes from 1036 m to 2523 m in Yunnan, China. The three collection sites of the new species are respectively located in catchments of the Jinsha River (the upper reaches of the Yangtze River), Nujinag River (the upper reaches of Salween River), and Lancang River (the upper reaches of Mekong River), suggesting that *B.borealis* sp. nov. may be common in the Three Parallel Rivers of Yunnan Protected Areas.

### ﻿ Branchiobaetis megasinus sp. nov.

Taxon classificationAnimaliaEphemeropteraBaetidae

EB6CF9E7-2D26-5178-B0A3-942A86F283EB

https://zoobank.org/59F827E6-DFA2-4020-9B44-36480650CD3F

Figs 8 , 9 , 10 , 11 , 12 , 13 , 14 , 15 , 16 , 17

#### Type material.

***Holotype*.** China • male larva in alcohol (mature); Guangdong, Shenzhen, Wutongshan River (22.5972°N, 114.2067°E); 30.xii.2023–1.i.2024; leg. Zhiheng Zhou. ***Paratypes*** (in alcohol): • 9 mature larvae, locality and date as holotype • 3 larvae, 1 male imago (reared specimen) on slide; Tai Po Kau Forest Stream, Hong Kong; 25.ii.1999; leg. Xiaoli Tong; 1 larva (on slide); Tai Po Kau Forest Stream, Hong Kong; 19.xi.1996; leg. Xiaoli Tong; 1 larva; Tai Po Kau Forest Stream, Hong Kong; 26.ii.1997; leg. Maria Salas; 1 larva (on slide); Shing Mum, Hong Kong; 7.i.1997; leg. Xiaoli Tong • 2 larvae; Ma Po Mei, Hong Kong; 10.x.1997; leg. Xiaoli Tong; 1 larva (on slide); Ma Po Mei, Hong Kong; 7.iii.1998; leg. Xiaoli Tong; 1 larva (on slide); Mt. Nankunshan, Longmen County, Guangdong; 16.ix.1994; leg. Xiaoli Tong • 3 larvae (1 on slide), Mt. Luofushan, Boluo County, Guangdong; 31.x.2023; leg. Xiaoli Tong, Zhiheng Zhou & Bangyi Wu • 5 larvae (1 on slide); upper reaches of Liuxihe River, Conghua, Guangzhou, Guangdong; 23–24.iii.2024; leg. Zhiheng Zhou & Bangyi Wu.

#### Description.

**Figure 8.**
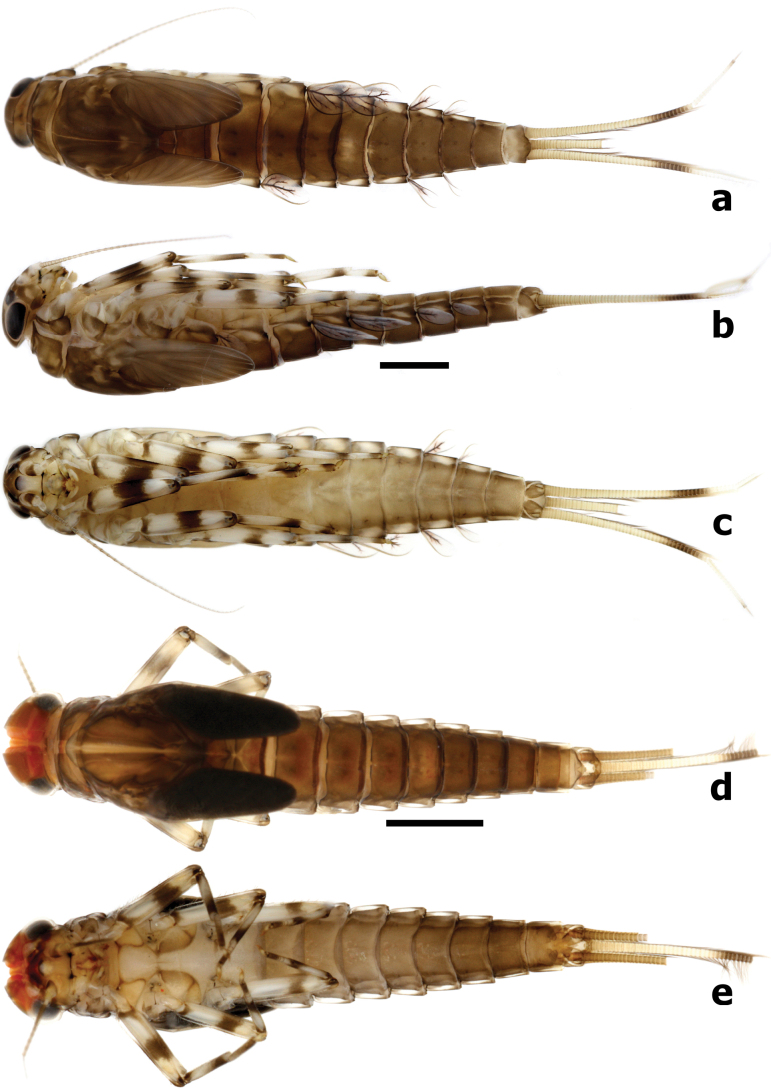
*Branchiobaetismegasinus* sp. nov. larval habitus **a** female larva (dorsal view) **b** female larva (lateral view) **c** female larva (ventral view) **d** final instar male larva (dorsal view) **e** final instar male larva (ventral view).

**Larva** (Fig. 8a–e). Body length (mm): female 7.0–8.5, male larvae slightly shorter than female, 6.0–7.5; antenna 3.0–4.0; cerci 3.0–4.0, paracercus ~ 4/5 length of cerci.

***Cuticular colouration*.** Body mainly brown or brownish green (in life) dorsally. Vertex uniformly brown. Antennal scape brown with off-white apex, pedicel off-white, flagellum pale brown. Pronotum mainly brown with irregular dark brown marks, meso- and metanotum mainly brown with irregular cream marks near base of forewing pads and a pair of small cream spots submedially. Legs contrasting bicoloured. Femur off-white with a large dark brown band medially and dark brown macula proximally and apically; tibia off-white with dark brown macula basally and distally; tarsus off-white with dark brown band apically. Abdominal tergites I–IV and VI–IX brown with a pair of cream stripes laterally; V brown with a cream, oval macula anterior medially and a pair of cream stripes laterally; X with yellow-brown shading to cream in the anterior portion and brown in posterior 1/2; tergites II–VIII with a pair of oblique dark brown medio-anterior sigilla and a pair of medioposterior sigilla (Figs 13e, 14a). Abdominal sternites with cream shading to pale brown posteriorly. Cerci cream to yellow-brown with dark brown bands medially and distally (Figs 8a–e, 15f); paracercus cream to yellow-brown with dark brown band near terminal; primary swimming bristles dark brown.

***Precursors of turbinate eyes*** (Figs 8d, e, 15a, b) in last instar male larvae normal, without elevated area with well-expressed facets.

**Figure 9.**
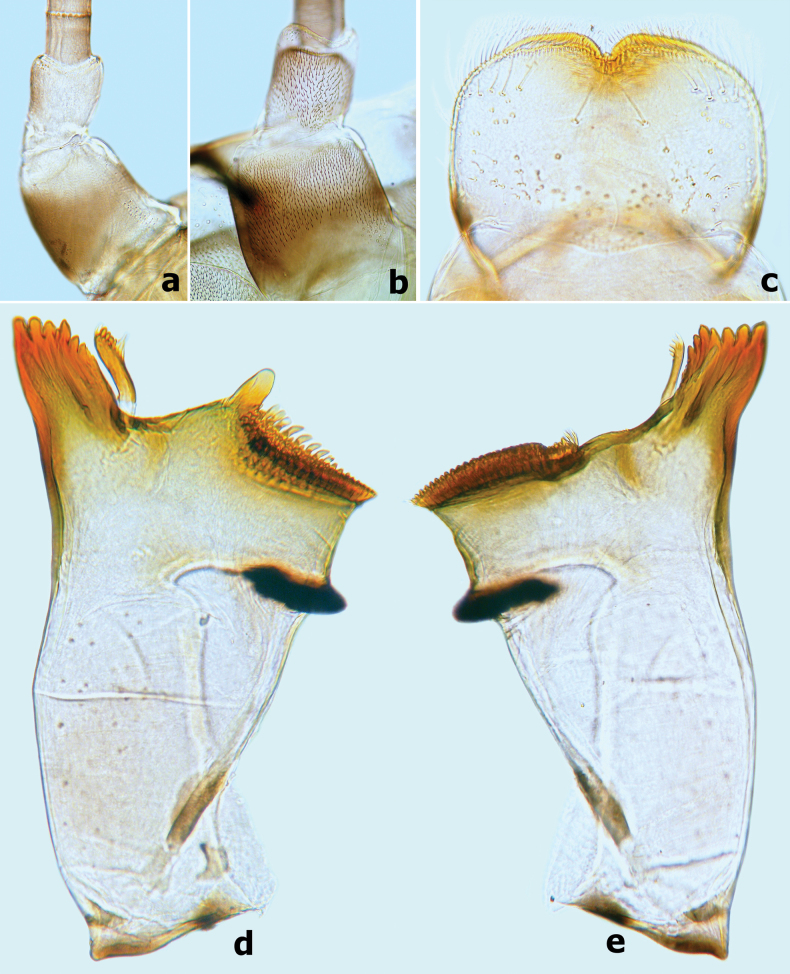
*Branchiobaetismegasinus* sp. nov. **a** antennal scape and pedicel **b** antennal scape and pedicel (final instar) **c** labrum **d** left mandible **e** right mandible.

***Antenna*** (Fig. 8a–c). Antenna ~ 3–4× of head width; scape smooth, without noticeable setae; inner margin of pedicel with tiny triangular denticles distolaterally (Fig. 9a, b).

***Labrum*** (Fig. 9c) nearly rectangular, width/length ratio ~ 1.5; anterior margin bordered with long and feathered setae and a median notch; dorsally with submedial pair of long, robust bristles and submarginal arc of six or seven long, robust bristles on each side of midline, several fine setae scattered proximally; ventral surface with densely fine setae medially and six or seven short, pointed setae laterally and disto-laterally.

***Left mandible*** (Fig. 9d). Incisor and kinetodontium fused; incisor with three denticles, kinetodontium with four main denticles decreasing in length and one additional minute denticle between incisor and kinetodontium; prostheca robust, apex with four bluntly denticles and two or three long, pointed denticles; margin between prostheca and mola straight with two or three fine, pointed minute spines; apex of mola without tuft of setae.

**Figure 10.**
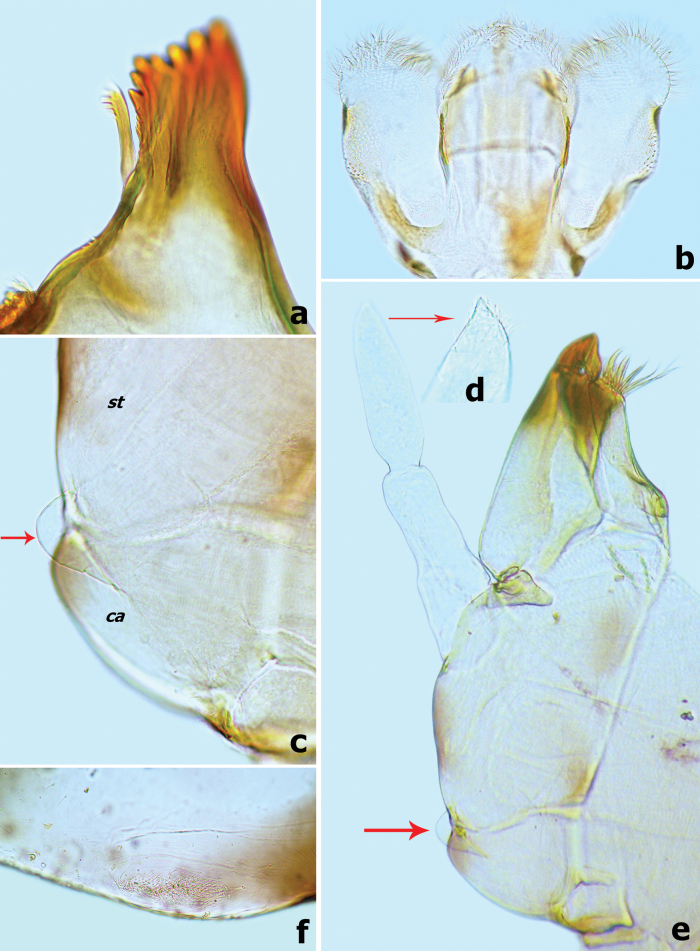
*Branchiobaetismegasinus* sp. nov. **a** apex of right mandible **b** hypopharynx and superlinguae **c** accessory gill between stipes and cardo of maxilla **d** apex of maxillary palp **e** maxilla **f** villopore of femur. Abbreviations: st: stipes, ca: cardo. Red arrows indicate the tongue-like accessory gill (**c, e**).

***Right mandible*** (Fig. 9e). Incisor and kinetodontium fused; incisor with three denticles; kinetodontium with four denticles, inner margin of innermost denticle with three small denticles (Fig. 10a); prostheca slender and toothbrush-like, with many sharp denticles on inner margin apically; margin between prostheca and mola slightly concave, occasionally with 1–3 fine, pointed minute spines; apex of mola with a tuft of straight setae.

***Hypopharynx and superlinguae*** (Fig. 10b). Lingua slightly longer than superlinguae, with numerous fine setae apically. Superlinguae distally rounded with numerous fine setae along apical margin.

***Maxilla*** (Fig. 10e). Galea-lacinia with three robust canines; crown of galea-lacinia with one regular row of 13–15 medium-size arcuate simple setae, second row compound of three dentisetae and row of 7–9 elongated pectinate setae; ventrally with two simple, apical setae under canines. Base of lacinia with one row of five or six long simple setae and one seta perpendicular to lacinia margin. Maxillary palp 2-segmented, longer than galea-lacinia, two segments subequal in length; apex of segment II with single tiny scale on small cone-shaped projection (Fig. 10d). Small tongue-like accessory gill located on outer side of the articulation between stipes and cardo (Fig. 10c, e).

**Figure 11.**
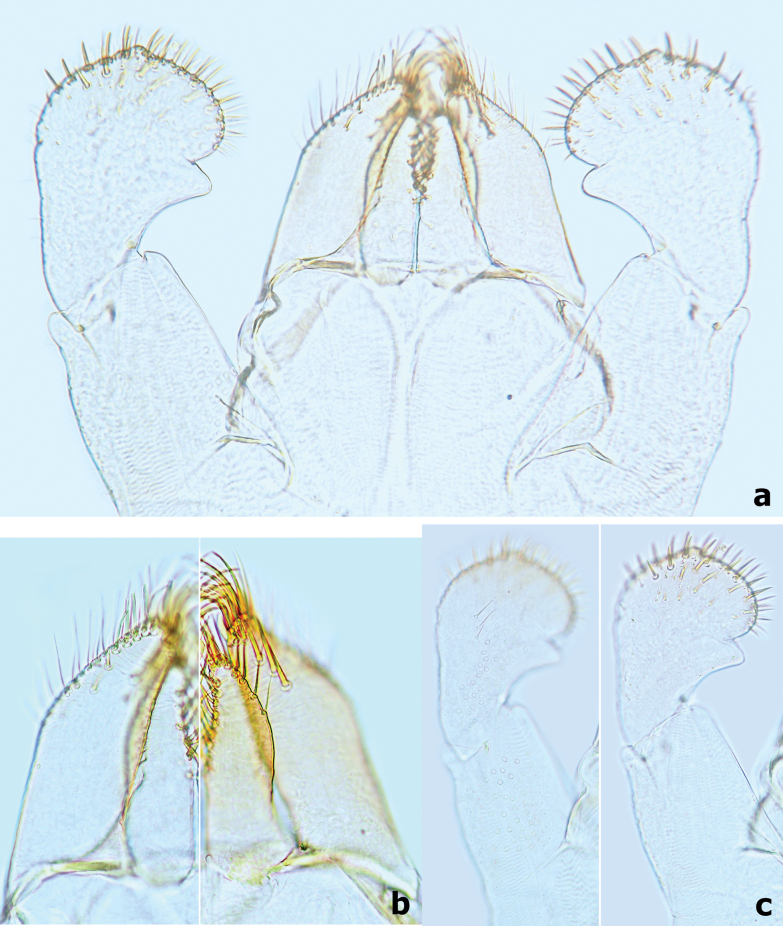
*Branchiobaetismegasinus* sp. nov. **a** labium **b** glossae and paraglossae (left: ventral view; right: dorsal view) **c** labial palp (left: dorsal view; right: ventral view).

***Labium*** (Fig. 11a). Glossae shorter and narrower than paraglossae, triangular with wide base, narrowing toward apex, inner margin of glossae with 7–10 long, simple setae and distal 1/3 of outer margin with ~ 5 long, simple setae, apex with three robust setae; paraglossae with two rows of long, robust, curved setae distoventrally, dorsal surface on distal 1/2 with one longitudinal row of two or three long, robust, spine-like setae near inner margin (Fig. 11b); labial palp 3-segmented, segment I longer than segments II and III combined, with many micropores dorsally; segment II triangular with distinct protuberance apico-laterally,~ 1.1× wider than base of segment III, dorsal surface with one longitudinal row of three medium simple setae and many micropores (Fig. 11c); segment III similar to asymmetrical onion-shaped dome, apex usually with a small cone-shaped projection, ventral surface covered with many spine-like, simple setae accompanied by fine setae distally (Fig. 11c).

***Hind wing pads*** well developed.

**Figure 12.**
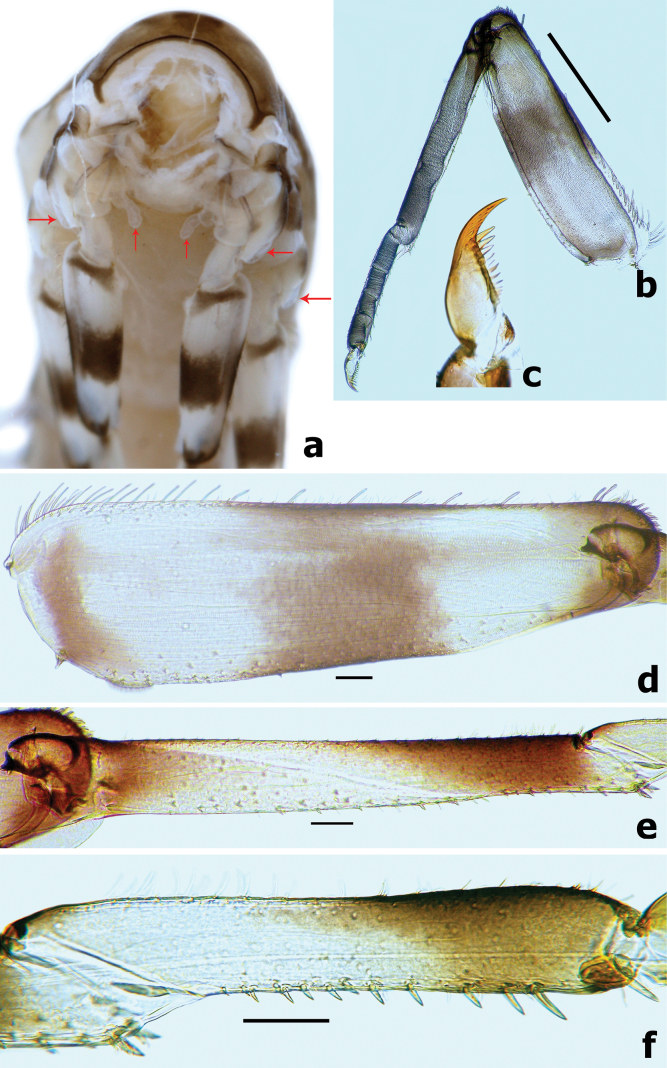
*Branchiobaetismegasinus* sp. nov. **a** accessory gills (vertical arrows) between coxa and prosternum on foreleg and bubble-like membranous swellings (horizontal arrows) **b** foreleg of final instar larva **c** claw **d** foreleg femur **e** foreleg tibia **f** foreleg tarsus. Scale bars: 0.5 mm (**b**); 0.1 mm (**d, e, f**).

***Forelegs*** (Fig. 12b–f). Ratio of foreleg segments (femur to claw) 6.9:5.9:3.5:1.0. *Femur*. Length ~ 3.5× maximum width. Dorsal surface covered with numerous crescent scale bases accompanied by fine setae; ventral face with sparse crescent scale bases and fine setae. Outer margin with two rows of different type setae: one row of 10–13 long, robust, blunt pointed setae and proximal row of 13–17 slender long setae (Fig. 12d); additional row of small, decurved hook-like setae along dorsal margin; ventral margin with small, apically pointed or rounded, spatulate setae. Apex rounded with many short, stout, hook-like setae anteriorly, dorsoapical setal patch formed by two stout setae. Villopore present and well developed (Figs 10f, 12d). A finger-like accessory gill on inner side of coxal articulation (between coxa and prosternum); hyaline bubble-like membranous swelling between coxa and trochanter (Fig. 12a). *Tibia*. Outer margin with one row of small, apically decurved hook-like setae and fine setae; ventral margin with row of longer, stout spine-like setae. Tibio-patellar suture present (Fig. 12e). Both surfaces covered with crescent-shaped scale bases, apically oval or pointed spatulate setae alternating with hair-like setae. *Tarsus*. Outer margin with fine setae and one row of short, curved hook-like setae; ventral margin with one row of ~ 11 stout spine-like pointed setae increasing in length toward apex (Fig. 12f), apex with single robust, spine-like pointed seta. Both surfaces covered with crescent scale bases. *Claws* hooked (Fig. 12c), with one row of 11–13 acute teeth, subapical setae absent.

***Middle and hind legs*** similar to foreleg in structure except for lacking the finger-like accessory gills on base of coxa.

**Figure 13.**
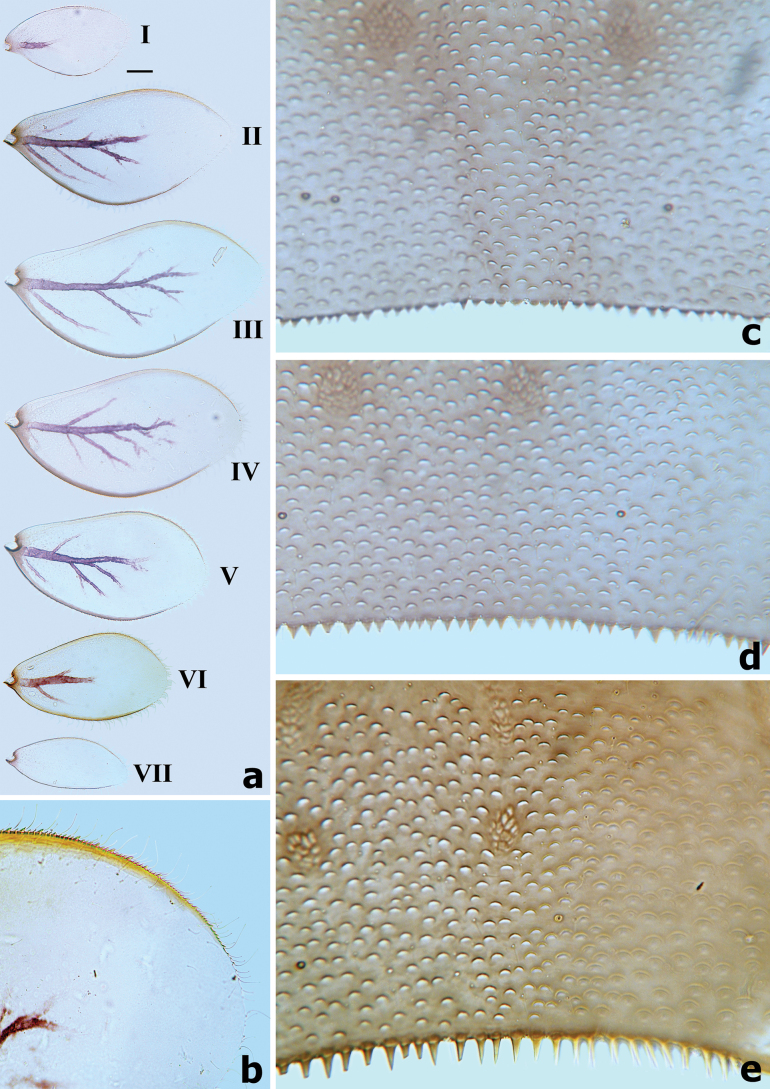
*Branchiobaetismegasinus* sp. nov. **a** gills I–VII **b** external margin of gill **c** posterior margin of abdominal tergite II **d** posterior margin of tergite IV **e** posterior margin of tergite VIII.

**Figure 14.**
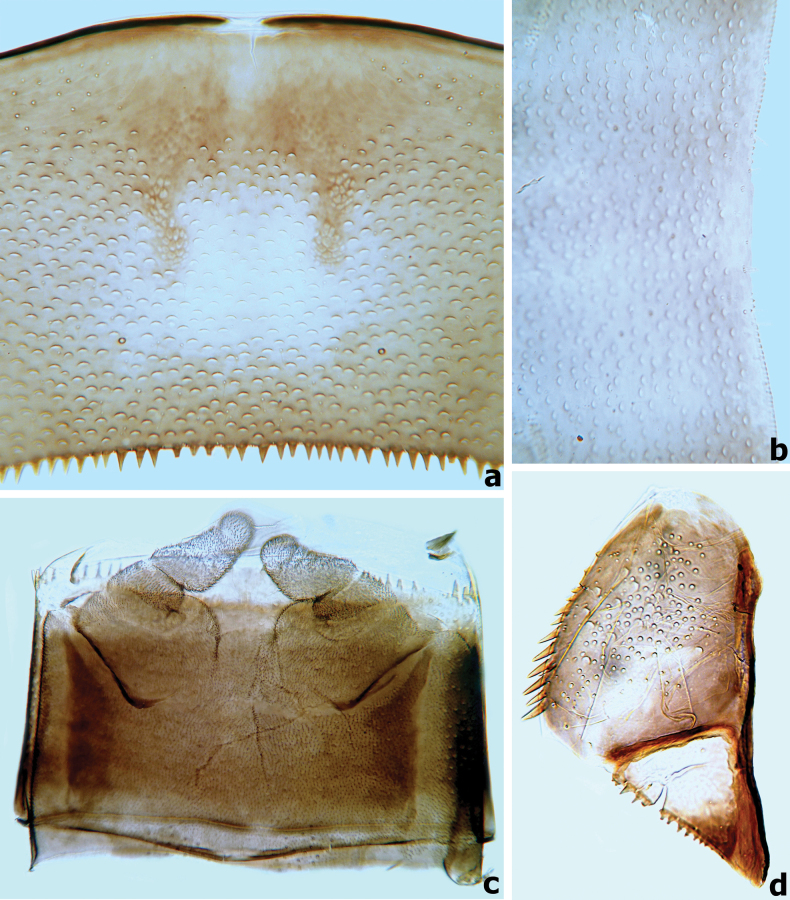
*Branchiobaetismegasinus* sp. nov. **a** middle part of abdominal tergite V **b** posterior margin of sternite VII **c** gonostyli bud under cuticle of last instar male larva **d** paraproct.

***Abdominal tergites and sternites*.** Both tergites and sternites densely covered with crescent scale bases and sparse hair-like setae, without any spatulate setae. Posterior margins of tergites I–X with triangular spines increasing in length from I to X (Figs 13c–e, 14a). Posterior margins of sternites I–IX smooth medially, but with row of dentate protuberances laterally (Fig. 14b).

***Gills*** (Fig. 13a) present on segments I–VII and well tracheated, ratio of gill length from I–VII = 1.0:1.8:2.0:1.9:1.6:1.3:1.0. External margins of all gills with small denticles intercalating fine hair-like setae (Fig. 13b), without any marginal spines or spatulate setae.

***Paraproct*** (Fig. 14d). Surface scattered with several crescent scale bases, fine setae and many micropores; margin with ~ 12 triangular spines; surface of cercotractor smooth, with 12–15 spines marginally.

**Figure 15.**
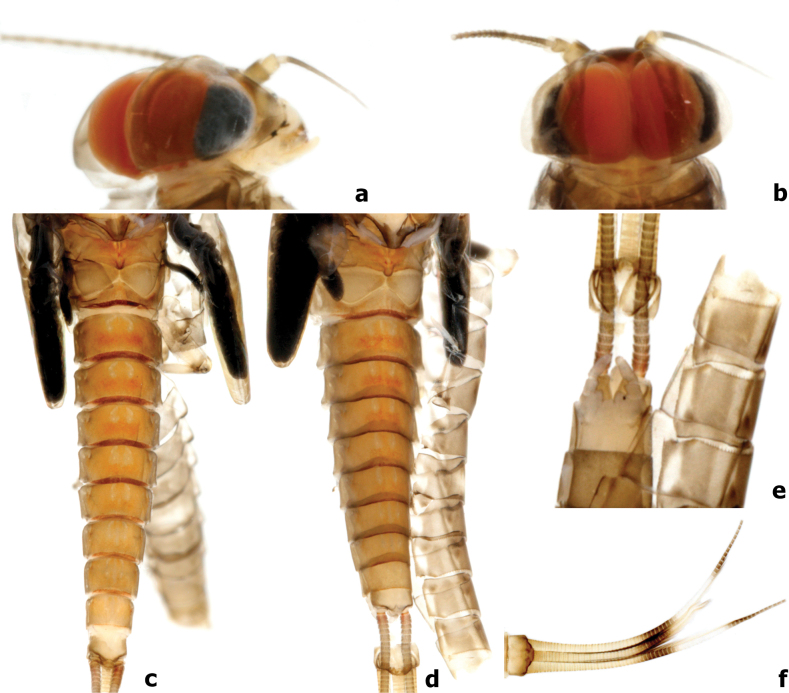
*Branchiobaetismegasinus* sp. nov. **a, b** head of final instar male larva before emergence **c** female abdomen extracted from last instar larva before emergence **d** male abdomen extracted from last instar larva before emergence **e** subimaginal gonostyli extracted from last instar larva **f** caudalii.

***Caudalii*** (Fig. 15f). Cerci with a row of swimming bristles on inner side of intersegment, each segment with row of pointed spines distally; paracercus with swimming bristles on both sides of intersegment.

***Gonostyli bud*** folded under cuticle of last instar larvae, segment II of gonostylus bud sharply bent towards middle and segment III sharply bent towards posterolaterally (Fig. 14c); subimaginal gonostyli extracted from last instar larva as Fig. 15e.

**Figure 16.**
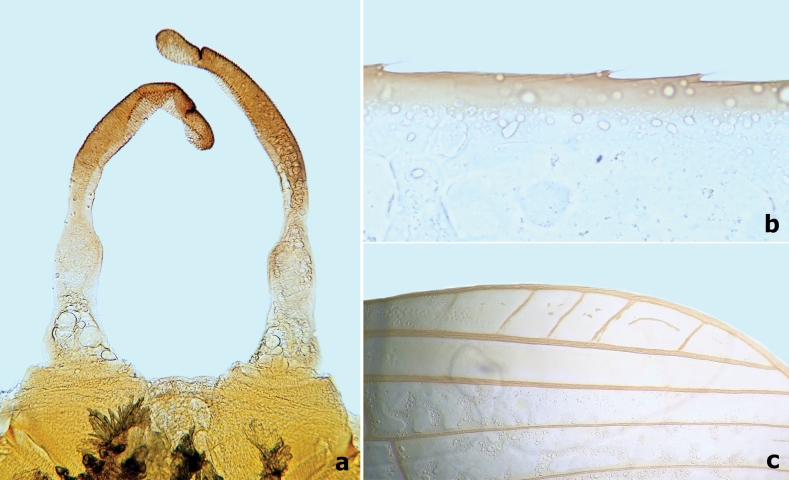
Male imago of *Branchiobaetismegasinus* sp. nov. **a** genitalia **b** costa of fore wing on basal portion **c** pterostigma area of fore wing.

**Figure 17.**
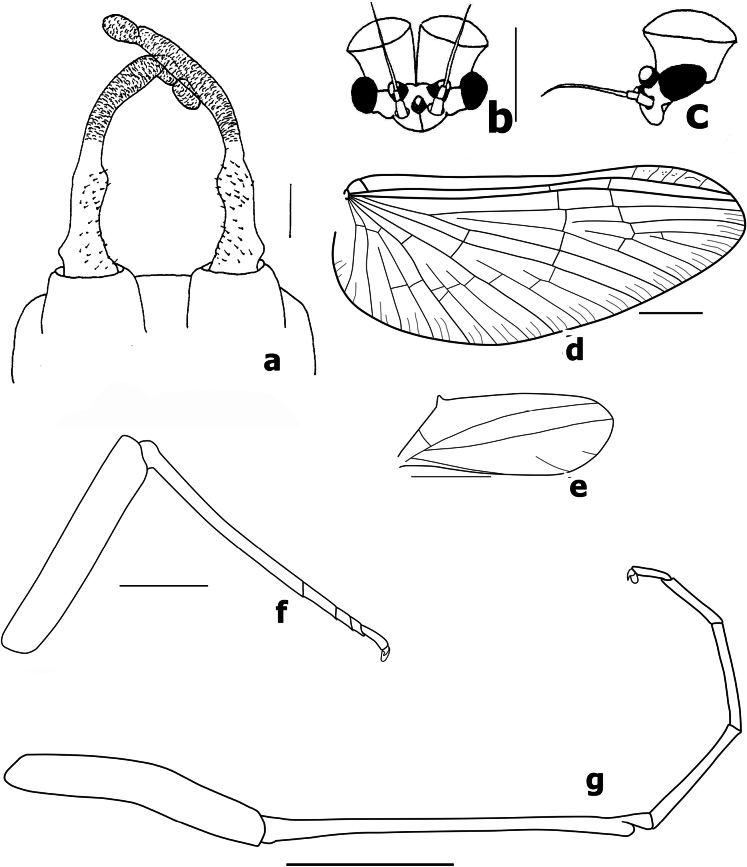
Male imago of *Branchiobaetismegasinus* sp. nov. **a** genitalia **b** head (anterior view,) **c** head (lateral view) **d** fore wing **e** hind wing (enlarge,) **f** hind leg **g** foreleg. Scale bars: 0.1 mm (**a**); 0.5 mm (**e, f**); 1.0 mm (**b, d, g**).

**Male imago.** Body length 6.4 mm. Forewing 6.3 mm. Cerci 18.0 mm. Turbinate eyes cylindrical (Fig. 17b, c), slightly widened apically, stalk and facetted surface orange; ocelli off-white with dark brown basal ring. Antennae longer than head capsule; flagella pale brown, pedicels and scapes cream. Pronotum pale with dark brown maculae medially; mesonotum pale to pale brown with brown markings medially and posterolaterally; metanotum yellow-brown to dark brown; thorax dark brown laterally. Forewings hyaline (Fig. 17d), longitudinal veins and paired marginal intercalaries yellow-brown, double intercalary veins longer than distance between corresponding longitudinal veins; costa serrated with pointed spines on basal portion (Fig. 16b), pterostigma area transparent washed pale yellow-brown, with four or five slanting cross veins (Figs 16c, 17d); hindwings (Fig. 17e) with acute costal process and three longitudinal veins. Fore femur pale, arched medially (Fig. 17g), fore tibia yellow-brown, fore tarsus pale yellow-brown; ratio of foreleg femur/tibia/tarsus = 1:1.4:1.4; ratio of foreleg tarsal segments =1.0:6.6:5.7:3.3:2.0; middle and hind legs similar to foreleg in colouration except with straight femora and apical spine on fused 1^st^+2^nd^ and 3^rd^ tarsal segments (Fig. 17f); all claws with one oval lobe and one pointed curved hook. Abdominal tergites I–VIII rust-red with anterior submedial pair of pale streaks, each with single purple-brown transverse streak along posterior margin, tergites IX–X pale. Genitalia (Figs 16a, 17a): unistyliger cylindrical, inner margin of segment I of gonostylus with distomedial swelling and outer margin with protuberance basally (Fig. 17a), segment III of gonostylus oblong. Cerci grey-white with rust tints basally.

**Female imago.** Unknown.

#### Etymology.

The specific epithet is a combination of *mega*- (derived from the Greek, meaning huge, large) and *sinus* (from Latin masculine adjective meaning bay or gulf). Thus, the name refers to the fact that the type series of the new species was found from the Guangdong-Hong Kong-Macao Greater Bay Area (Greater Bay Area).

#### Distribution

**(Fig. 23).** China: Guangdong (Shenzhen, Guangzhou, Boluo, Longmen) and Hong Kong (Tai Po Kau Forest Stream, Shing Mun, Chuen Lung, Ma Po Mei, Ng Tung Chai, Mui Tsz Lam, Shek Mun Kap).

#### Larval habitat

**(Fig. 22a).** The species usually live in moderately rapid, well-aerated riffles at low-altitude (< 300 m a.s.l.) forest streams with gravel and cobble substrates. The physicochemical parameters of the type locality (Wutongshan River, Shenzhen in December) are as follows: river width 5–7 m, water depth 10–20 cm, water temperature 18.3 °C, current velocity 0.25 m/s, DO 9.4 mg/l, pH 8.0 and TDS 43.9 mg/l.

### ﻿ Megabranchiella longusa

Taxon classificationAnimaliaEphemeropteraBaetidae

Phlai-ngam & Tungpairojwong, 2022

7B0507CD-1453-55E5-BE24-83370FC43629

**Figure 18.**
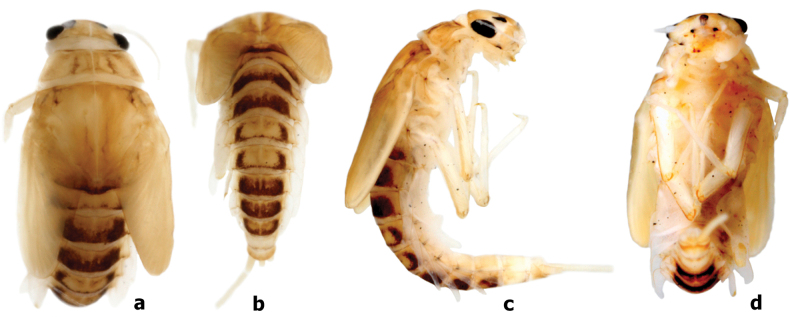
Larval habitus of *Megabranchiellalongusa***a, b** female larva (dorsal view) **c** female larva (lateral view) **d** female larva (ventral view).

**Figure 19.**
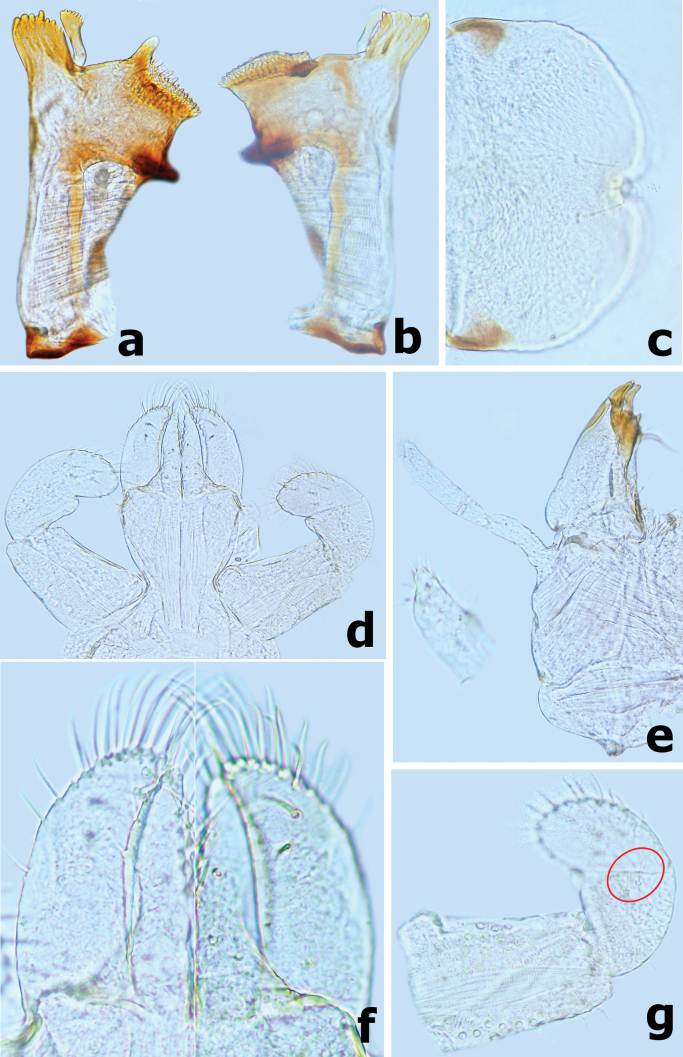
*Megabranchiellalongusa***a** left mandible **b** right mandible **c** labrum **d** labium **e** maxilla **f** glossae and paraglossae (left: dorsal view; right: ventral view) **g** labial palp (dorsal view). Red ellipse encloses dorsal setae.

**Figure 20.**
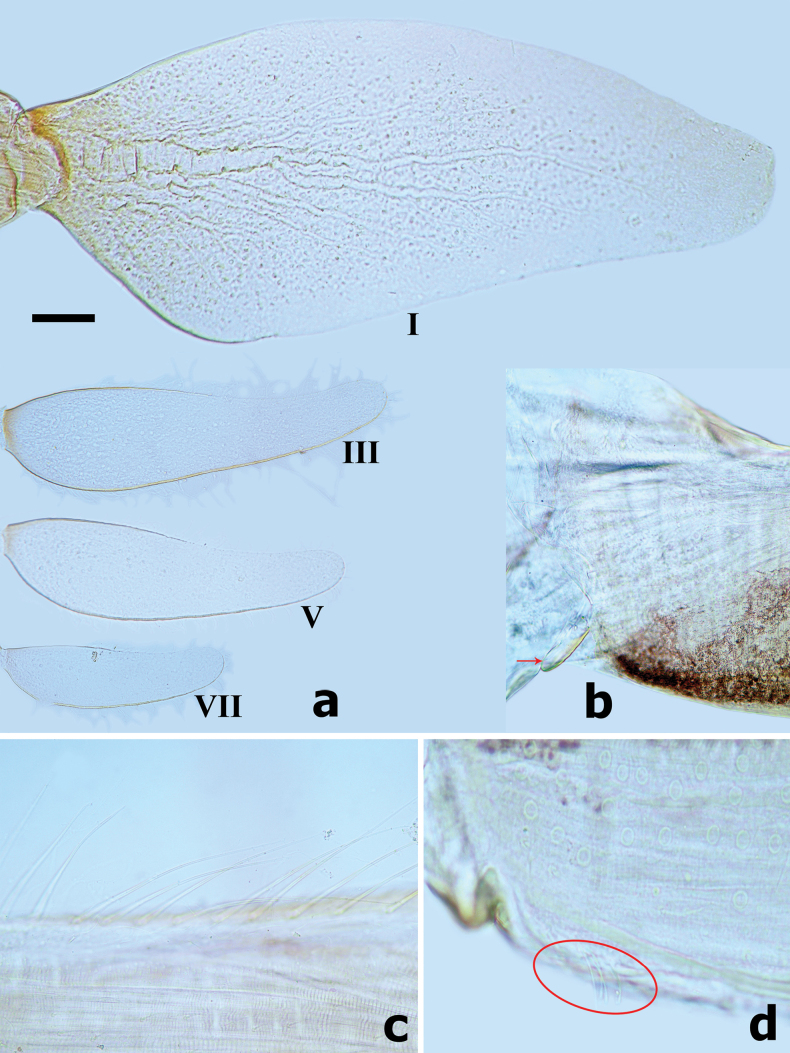
*Megabranchiellalongusa***a** gills I, III, V, VII **b** hindwing pad **c** dorsal margin of femur **d** villopore of femur. Red arrow indicates hindwing pad. Red ellipse encloses villopore . Scale bar: 0.1 mm.

**Figure 21.**
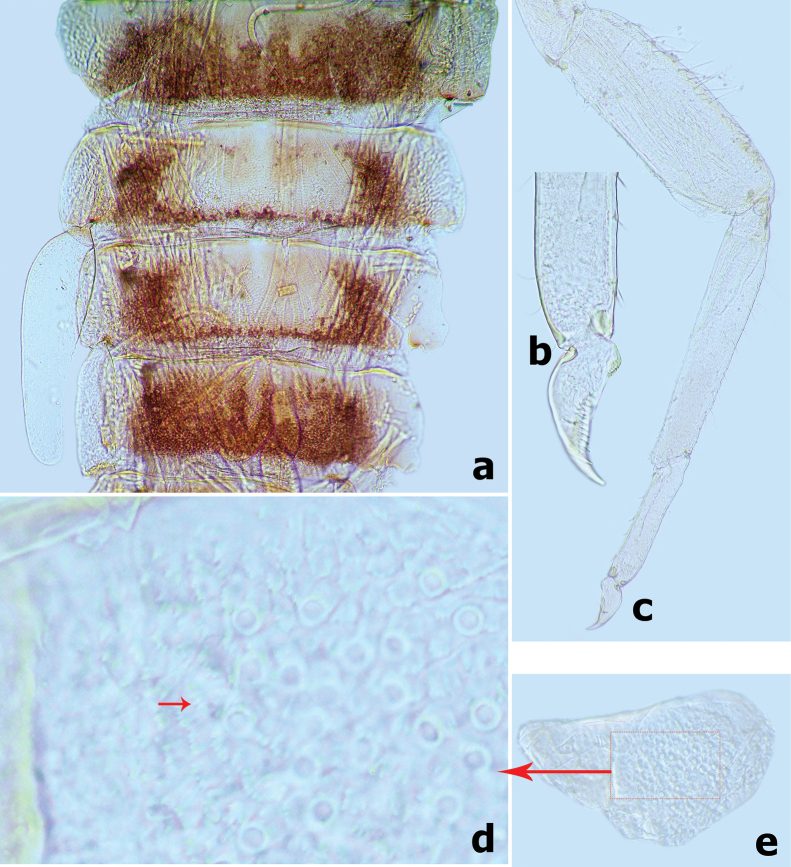
*Megabranchiellalongusa***a** abdominal tergites III–VI **b** claw **c** foreleg **d** notched scales on paraproct surface **e** paraproct.

Figs 18 , 19 , 20 , 21

Megabranchiella longusa : Phlai-ngam & Tungpairojwong in Phlai-ngam et al. 2022: 16.

#### Material examined.

One female larva on slide; Yunnan, Lushui, Bajiao River (a tributary of the Nujiang River, altitude 1112 m); 21.iii.2019; leg. Xiaoli Tong.

#### Diagnosis.

Female larva (Fig. 18a–d), body short and flattened, length ~ 4.0 mm; body colour pattern as Fig. 18a–c. ***Head*.** Labrum nearly semicircular, ~ 1.4× wider than long; anteromedian notch deep with a small, rounded lobe at base; dorsal surface in distal 1/2 with one pair of long, simple setae near midline and irregular row of three medium, simple setae (Fig. 19c). Left mandible (Fig. 19a), incisor and kinetodontium fused, incisor with four denticles, kinetodontium with six denticles decreasing in length, prostheca robust, apex with six bluntly denticles and one or two long, spine-like denticles; incisor of right mandible with four denticles, kinetodontium with four denticles, inner margin of innermost denticle with row of small denticles; prostheca robust, apex with comb-like structure, with many denticles apically (Fig. 19b). Maxilla (Fig. 19e), galea-lacinia of with three robust canines, base of lacinia with one row of four long, simple setae and one seta perpendicular to lacinia margin; maxillary palp 2-segmented, apex of segment II with a small cone-shaped projection. Labium (Fig. 19d, f), glossae shorter and narrower than paraglossae, paraglossae with three rows of long curved setae distoventrally, dorsal surface on distal 1/2 with one longitudinal row of two or three long, spine-like setae near inner margin; labial palp 3-segmented, segment I longer than segments II and III combined, segment II triangular with small protuberance apico-laterally, dorsal surface of segment II with row of two robust, simple setae near distal margin (Fig. 19g). ***Thorax*.** Hindwing pads reduced (Fig. 20b). Forelegs (Fig. 21c), femur with a row of long, robust, pointed setae along dorsal margin, surface with notched scales anteromedially (Fig. 20c), villopore present (Fig. 20d); tibia with a row of long, pointed setae and short, blunt spatulate setae, tibio-patellar suture present; ventral margin of tarsus with one row of four robust, spine-like setae; claws hooked (Fig. 21b) with one row of 13 acute teeth, subapical setae absent. Middle and hind legs similar to foreleg in structure. ***Abdomen*.** Abdominal tergites and sternites with smooth posterior margins (Fig. 21a); gills present on abdominal tergites I–VII, gill I oriented ventrally, extremely enlarged and elongated (Fig. 20a), covering abdominal sternites II–VI (Fig. 18c), gills II–VII oriented dorsolaterally, elongated oval similar to tongue blade (Figs 18c, 20a), gill margins with long, fine, hair-like setae, ratio of gill length from I–VII = 3.2:1.7:1.7:1.7:1.5:1.3:1.0; paraproct with smooth margin, without marginal spines or spatulate setae, surface with micropores and patch of notch scales (Fig. 21d, e).

#### Distribution

**(Fig. 23).** Thailand (Chiang Mai and Nan Provinces) and China (Yunnan Province).

#### Larval habitat

**Figure 22.**
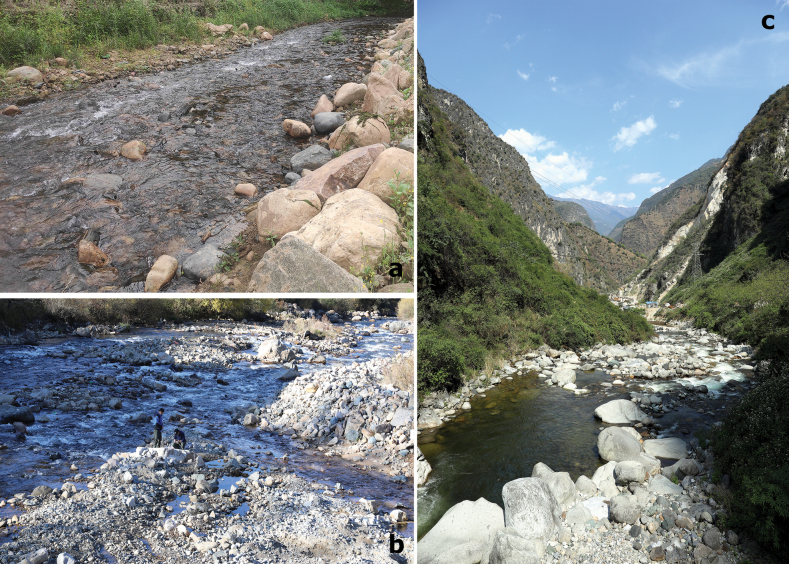
Representative sites of larval habitat **a***Branchiobaetismegasinus* sp. nov. Upper reaches of Liuxihe River, Conghua, Guangdong **b***Branchiobaetisborealis* sp. nov. Lapu River, Weixi, Yunnan **c***Megabranchiellalongusa* Phlai-ngam & Tungpairojwong. Bajiao River, Lushui, Yunnan.

**(Fig. 22c).** The species was collected in a swift, unshaded stream with cobble substrate at an altitude of ~ 1100 m in Yunnan, China.

**Figure 23.**
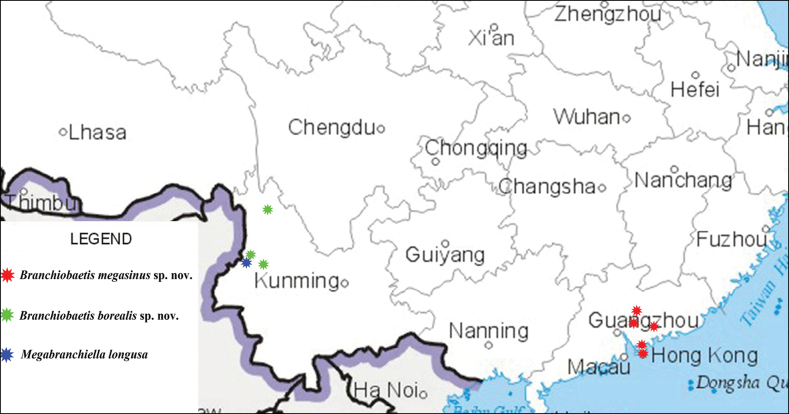
Distribution map of the genera *Branchiobaetis* and *Megabranchiella* in China.

#### Remarks.

Geographically, this record represents the farthest distribution north of the genus *Megabranchiella* so far. We expect that more species of the genus will be discovered with the expansion of the investigation range in China.

## ﻿Discussion

The morphological characters of *B.borealis* sp. nov. and *B.megasinus* sp. nov. are slightly different from other known species of the genus by the following characters in the larval stage: (1) antennal scape smooth without spatulate setae, inner margin of pedicel with tiny triangular denticles distolaterally; (2) mandible without blade-like incisor; (3) maxillary accessory gill reducing to small tongue-like structure. However, the presence of the following combination of characteristics confirms that they belong to the genus *Branchiobaetis*: (1) one finger-like accessory gill ventrally on coxal articulation of foreleg; (2) accessory gill outside laterally between stipes and cardo at the base of maxillae; (3) bubble-like membranous swelling between coxa and trochanter of legs; (4) one row of row of small, apically decurved hook-like setae along dorsal margin of femur, tibia, and tarsus on legs. In addition to these three differences, the new species can be distinguished from other known species of *Branchiobaetis* by the following combination of characters in the larvae.

In *B.borealis* sp. nov.: (1) abdominal tergites with contrasting colour pattern as Fig. 1; (2) paraglossae of labium with three rows of long, robust, distally pectinate setae distoventrally; (3) femur with row of long, stout, clavate setae along dorsal margin, villopore well developed on all legs; (4) claw without subapical seta; (5) untypical folding way of gonostyli bud.

In *B.megasinus* sp. nov.: (1) abdominal tergites almost uniformly brown except for with a cream oval macula anterior medially on tergite V, legs with contrasting cream and brown alternating bands as Fig. 8; (2) paraglossae of labium with two rows of long, robust, distally pectinate setae distoventrally; (3) femur with row of long, stout, blunt pointed setae along dorsal margin, villopore well developed on all legs; (4) claw without subapical seta.

*Branchiobaetismegasinus* sp. nov. is the second species in the genus that is known with a male imago. Compared with male imago of *Branchiobaetisjavanicus* (Ulmer, 1913) (in Kaltenbach et al. 2022b), the new species can be distinguished by the following combination of characters: (1) precursors of turbinate eyes are normal based on the exuviae of last instar male larva (vs with elevated area with well-expressed facets in *B.javanicus* (Kaltenbach et al. 2022b: fig. 5c); (2) forewing costa serrated with pointed spines on basal portion (Fig. 16b), pterostigma area almost transparent with four or five– slanting cross veins (vs costa smooth and pterostigma area brown with at least ten slanting cross veins or veinlets in *B.javanicus*); (3) femur of foreleg arched medially (Fig. 17g). In *B.javanicus*, the femur is straight, but the tibia is slightly arched medially (Kaltenbach et al. 2022b: fig. 8a); (4) genitalia: inner margin of segment I of gonostylus with distomedial expansion and outer margin with protuberance basally (Fig. 17a), segment III of gonostylus oblong (vs segment I of gonostylus with projected blunt angle proximad of its middle; segment III short and triangular in *B.javanicus*). Consequently, as Kaltenbach et al. (2022b) pointed out, the imaginal generic diagnosis is currently still difficult to define until more male imagos are described.

The most striking characteristic of *Branchiobaetis* is the presence of the accessory gills (the etymology of the genus is derived from this character), i.e., coxal gills (located between coxae and prosterna) and maxillary gills (located between stipes and cardo). An overview of accessory gills among mayflies and a discussion of the possibility of their respiratory function is given by Kaltenbach et al. (2022b). Other than that, another interesting character of the genus is the presence of a bubble-like, membranous swelling located between the coxa and trochanter and between the coxa and pleurite, and the branches of tracheae are observed under the swelling. These thin-walled, air sac-like structures on the articulation of legs are rare in Baetidae, even in Ephemeroptera. It is unclear whether they serve as the accessory respiratory organs, or the air sacs are formed by tracheal dilatation for adjusting their respiration or buoyancy (Harrison et al. 2023).

In aquatic ecosystems, dissolved oxygen (DO) concentration is not only the primary limiting factor that determines aquatic insect physiology and behaviour, but also a critical measure of habitat quality and river health. Thus, DO concentrations are one of major factors influencing biological diversity and productivity of freshwater ecosystems worldwide (Dudgeon 2008; Jacobus et al. 2019). According to our rearing experience, when transferring the larvae of the new species together with other baetid larvae (e.g., *Baetis* spp., *Labiobaetis* sp., *Liebebiellavera*) to indoors for rearing, we found that the mortality rate of *B.borealis* sp. nov. and *B.megasinus* sp. nov. was much higher than those of other baetid larvae without such accessory gills, even with the aid of portable air pump during transportation. In addition, the same is true of *Branchiobaetisjavanicus* (Ulmer, 1913) in Southeast Asia as the “larvae are unable to live for a long time in stagnant water” (Kaltenbach et al. 2022b). One possible explanation is that they are possibly a sensitive species to low dissolved oxygen or hypoxia owing to their relatively high oxygen demand in water. In present study, the two new species occur most commonly in very clean, cool, and well-oxygenated mountainous or forest streams in subtropical China. For example, the dissolved oxygen concentration is up to 9.4 mg/L in the type locality of *B.megasinus* sp. nov. In general, as the water temperature increases, the dissolved oxygen concentration decreases. Thus, the average water temperature of rivers in Southeast Asia is usually warmer than that of rivers in subtropical China (Dudgeon 2008). The accessory gills on maxillae of the new species are less developed and reduced to a small tongue-like structure (Figs 3b, 10c). In contrast, the maxillary gills of the species in Southeast Asia are well developed (Kaltenbach et al. 2022b: figs 1a, 18j), which may be an adaptation to low dissolved oxygen environment in Southeast Asia, as only by increasing the absorption area of maxillary gills can they absorb enough dissolved oxygen in water. Nevertheless, it remains unclear if the contribution of coxal gills or maxillary gills to oxygen uptake is significant or negligible. The establishment of these two new species will facilitate the subsequent in-depth studies on their morphological anatomy, oxygen uptake, and other biological traits.

## Supplementary Material

XML Treatment for Branchiobaetis borealis

XML Treatment for Branchiobaetis megasinus

XML Treatment for Megabranchiella longusa

## ﻿Additional information

### Author ORCIDs

Xiaoli Tong https://orcid.org/0000-0003-1731-229X

Zhiheng Zhou https://orcid.org/0009-0009-0763-8713

Bangyi Wu https://orcid.org/0009-0005-6398-5243

### Data availability

All of the data that support the findings of this study are available in the main text.
